# The Moderating Effect of Comfort from Companion Animals and Social Support on the Relationship between Microaggressions and Mental Health in LGBTQ+ Emerging Adults

**DOI:** 10.3390/bs11010001

**Published:** 2020-12-23

**Authors:** Angela Matijczak, Shelby E. McDonald, Camie A. Tomlinson, Jennifer L. Murphy, Kelly O’Connor

**Affiliations:** 1 School of Social Work, Virginia Commonwealth University, Richmond, VA 23284, USA; smcdonald3@vcu.edu (S.E.M.); tomlinsonc2@vcu.edu (C.A.T.); murphyjl4@vcu.edu (J.L.M.); 2 Department of Psychology, Virginia Commonwealth University, Richmond, VA 23284, USA; oconnorke2@vcu.edu

**Keywords:** LGBTQ, microaggressions, mental health, companion animals, social support, minority stress, human–animal interaction

## Abstract

LGBTQ+ (lesbian, gay, bisexual, transgender, queer, and other sexual/gender minority identities) individuals frequently report exposure to microaggressions, which are associated with deleterious mental health outcomes. Social support from humans has been found to be an important protective factor for LGBTQ+ emerging adults. However, an underexplored area of research is the protective role of interactions with companion animals for this population. We conducted simple and multiple moderation analyses to explore whether and to what extent emotional comfort from companion animals and human social support moderated the relationship between LGBTQ-related microaggressions and depressive and anxiety symptoms. Our sample included 134 LGBTQ+ emerging adults (mean age of 19.31). We found that social support moderated the relationship between microaggressions and depressive symptoms. The relationship between microaggressions and depressive symptoms was not significant at high levels of social support, indicating the protective nature of human social support. Comfort from companion animals also moderated the relationship between interpersonal microaggressions and depressive symptoms. For participants with high or medium levels of emotional comfort from companion animals, interpersonal microaggressions were positively associated with depressive symptoms. Our results highlight the need to further investigate the complex role of relationships with companion animals on mental health outcomes for LGBTQ+ emerging adults.

## 1. Introduction

Individuals who identify as LGBTQ+ (lesbian, gay, bisexual, transgender, queer, and other sexual or gender minority identities) are a diverse group of people that face increased risk of deleterious mental health outcomes due to experiences of victimization, discrimination, and other forms of sexual and gender minority stress [1,2,3,4,5]. The term sexual minority is often used to refer to individuals whose sexual orientation falls outside the scope of the dominant culture of heteronormative sexuality; examples include, but are not limited to, men who have sex with men; women who have sex with women; pansexual, bisexual, asexual, and queer-identified people. In the current work, the term gender minority is used to refer to those whose gender identity and/or expression does not align with or conform to societal expectations (perceived by others as a gender expression not typical of their biological sex), such as transgender, gender expansive, and non-binary-identified people [6]. The Minority Stress Model [7] is a conceptual framework that has been applied to explain the unique stressors LGBTQ+ individuals experience and how these stressors impact risk and resilience in LGBTQ+ individuals and communities. In this framework, Meyer [7] distinguishes between two different types of stressors: distal stressors and proximal stressors. Distal stressors, such as prejudice and discrimination, are external and objective in nature. In contrast, proximal stressors are subjective, personal appraisals or perceptions, such as internalized homophobia and other negative societal attitudes associated with one’s own identity. 

One distal stressor that LGBTQ+ individuals frequently experience is microaggressions [8,9]. Microaggressions are typically unconscious behaviors or statements directed at members of marginalized groups that reflect a hostile or discriminatory message [10,11,12]. For example, interpersonal LGBTQ-related microaggressions include the use of heterosexist or transphobic language in humor and the fetishization of LGBTQ+ people and/or their relationships [13,14,15]. Additionally, LGBTQ+ people may experience environmental microaggressions, such as exposure to heterosexist media or a lack of gender-inclusive bathrooms in public buildings [10,16]. 

Prior research suggests that microaggressions are important forms of discrimination with particularly negative impacts on LGBTQ+ young people. In a sample of LGBQ emerging adults, Woodford et al. [8] found that interpersonal and environmental microaggressions predicted psychological distress and low self-acceptance, whereas neither blatant discrimination nor victimization was significantly related to either outcome. Similarly, results from other studies have found links between experiences of racial and/or LGBTQ-related microaggressions and a host of detrimental outcomes, including higher rates of depressive, anxiety, or posttraumatic stress symptoms [17,18,19,20,21]; lower self-esteem and self-efficacy [21,22,23,24]; and negative perceptions of one’s own LGBTQ+ identity [24].

### 1.1. Social Support as a Protective Factor

Given the myriad of negative outcomes associated with experiences of LGBTQ-related microaggressions, it is vital that researchers identify factors that attenuate those relations and promote mental health among LGBTQ+ individuals. There is robust evidence of the relationship between various domains of social support (e.g., support derived from family relationships, peers, schools) and psychological wellbeing in LGBTQ+ emerging adult populations. For example, studies indicate that LGBTQ+ adolescents and emerging adults who have supportive relationships with family members report fewer depressive and anxiety symptoms [25,26,27]. Additionally, qualitative researchers who have examined the impact of intersectional microaggressions on LGBTQ+ people of color suggest that participants seek out social support and establish supportive networks as a method of coping with their experiences of microaggressions [28,29,30].

There is emerging quantitative evidence that social support may serve as a protective factor that buffers the relationship between LGBTQ-related minority stressors, such as victimization and discrimination, and negative mental health outcomes [31,32,33,34]. However, few studies have specifically investigated the moderating role of social support on the relationship between microaggressions and mental health outcomes in LGBTQ+ emerging adult (aged 18-25 years) populations. To our knowledge, only two studies have explored this moderation model: one study examined identity-affirming social support with a Dutch sexual minority population [35], and the other investigated support from peers with a sample of sexual minority individuals with disabilities [36]. Although neither of these studies found that social support significantly moderated the relationship between sexual minority microaggressions and mental health outcomes, it is important to consider that there are many sources of social support that have not yet been investigated in this area of research (e.g., support derived from relationships with companion animals). Further studies are needed to explore the relationship between social support, microaggressions, and mental health among U.S. LGBTQ+ populations.

### 1.2. Relationships with Companion Animals as a Protective Factor

One underexplored area in research with LGBTQ+ populations is the potentially beneficial relationship between LGBTQ+ individuals and their companion animals. There is a large body of evidence that suggests that companion animals, particularly dogs and cats, may serve as an important source of social support for youth and emerging adults [37,38]. In a study of university students, Meehan et al. [39] found that participants perceived their companion animals as a form of social support distinct from support derived from family members, friends, or significant others. Further, social support derived from companion animals was ranked significantly higher than social support derived from friends (but not family or significant others), suggesting that relationships with companion animals are uniquely meaningful compared to other relationships. Additionally, youth and emerging adults report seeking out their dogs and cats for emotional comfort during stressful and traumatic situations [40,41,42]. However, these studies relied on binary categories of gender identity and did not collect information on the participants’ sexual orientation; thus, it is difficult to generalize these findings to the unique developmental contexts of sexually and gender-diverse populations.

More than 65% of LGBTQ+ adults report owning pets [43,44]. Several qualitative studies have investigated the impact of pet ownership among LGBTQ+ older adult samples and provide evidence that companion animals serve as sources of unconditional love, emotional comfort, and belongingness [45,46,47]. A quantitative study conducted by Muraco et al. [48] found that pet-owning LGBTQ+ older adults reported significantly higher perceived social support than LGBTQ+ older adults without pets. However, less is known about the role of companion animals in the lives of younger LGBTQ+ populations and the potential protective impact of relationships with companion animals on experiences of LGBTQ-related stressors. Recent studies conducted with LGBTQ+ adults experiencing family violence found that pet ownership and positive interactions with pets buffered the relationship between victimization by family members and psychological stress [49,50]. Additionally, a recent study found an indirect effect of exposure to interpersonal microaggressions on personal hardiness via human-animal interaction; increases in interpersonal microaggressions were related to increases in human–animal interaction, which in turn were related to increases in self-reported personal hardiness [51]. This is the only study, to our knowledge, to investigate the role of pets in the relationship between microaggressions and psychological outcomes. However, no study, to our knowledge, has investigated the moderating role of comfort from companion animals on the relationship between microaggressions and mental health outcomes with an LGBTQ+ emerging adult population. 

### 1.3. The Current Study

**Figure 1:**
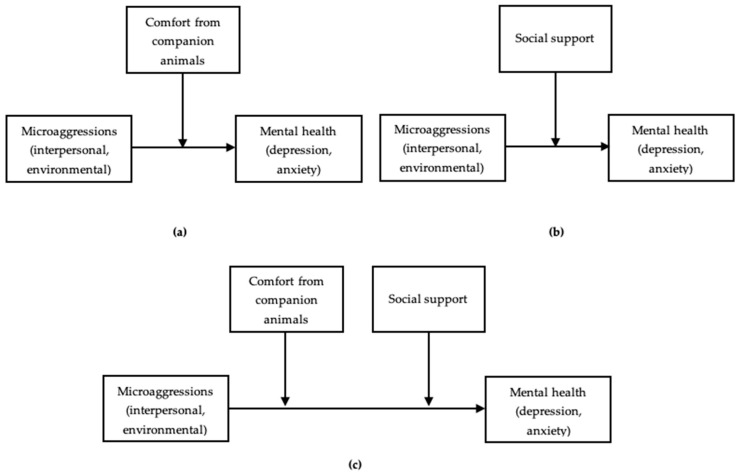
Conceptual models for moderation analyses. Covariates are not shown in the figures for clarity. (**a**) Simple moderation model with comfort from companion animals as the moderating variable; (**b**) simple moderation model with social support as the moderating variable; (**c**) multiple moderation model.

The current study addresses this gap in knowledge regarding the moderating role of social support and emotional comfort from companion animals on the relationship between LGBTQ-related microaggressions and mental health outcomes in an LGBTQ+ emerging adult population. Based on evidence from prior studies, we hypothesize that experiences of microaggressions will be associated with greater depressive and anxiety symptoms. Further, we hypothesize that emotional comfort from companion animals and social support from humans will each independently mitigate the deleterious effect of microaggressions on mental health outcomes across all models. We also hypothesize that LGBTQ+ emerging adults exposed to microaggressions who report high levels of both comfort from companion animals and social support from humans will experience the lowest levels of depressive and anxiety symptoms. Consequently, the objectives of this study were: (a) to test the moderating effect of emotional comfort derived from companion animals on the relationship between LGBTQ-related microaggressions (interpersonal and environmental) and depressive and anxiety symptoms (Figure 1a); (b) to test whether and to what extent human social support moderates the relationship between microaggressions and depressive and anxiety symptoms (Figure 1b); and (c) to explore whether, and to what degree, the relationship between microaggressions and depressive and anxiety symptoms varies as a function of emotional comfort derived from companion animals and social support derived from humans, when holding the other moderator constant (Figure 1c).

## 2. Materials and Methods

### 2.1. Participants

The participants in this study were a part of a larger, longitudinal study of LGBTQ+ youths’ experiences of stressors and supports. Due to the ongoing nature of our research, the current study employs a cross-sectional design using data from wave 1 of data collection. The inclusion criteria included self-identifying as LGBTQ+, being 15–21 years of age, and being able to understand spoken English. Due to our focus on the emerging adulthood period of development, we limited the sample in the current study to participants between the ages of 18 and 21 years who lived with a dog or cat within the past year (*n* = 138). Due to missing data, four participants were excluded from our analyses, decreasing our sample size to 134. A majority of participants identified as White (62.7%), and the average age was 19.31 years (*SD* = 1.11). Participants self-identified with a variety of LGBTQ+ identities: 98.5% reported a sexual minority identity and 49.5% identified as a gender minority (e.g., transgender, non-binary). Almost all (91%) of the participants reported current enrollment in school, with 64.1% having completed at least some portion of college requirements. Additional demographic information of our sample is provided in Table 1.

### 2.2. Procedures

Recruitment and data collection occurred between April 2019 and June 2020 in an urban city in the southeastern United States. The university’s Institutional Review Board approved the procedures for the current study (HM20014415). Participants were recruited in partnership with five local, community-based agencies that offer LGBTQ+ inclusive services to young people through the following means: (a) advertising our study through social media platforms, including Facebook and Twitter, and through local listservs; (b) posting study flyers in the community; (c) conducting information sessions at community organizations serving LGBTQ+ young people and their families; and (d) connecting with individuals through local, community LGBTQ+ events. Those interested in participating contacted the study coordinator via email or phone call. 

Screening interviews were conducted via phone call. If a participant met all the inclusion criteria, the study coordinator then scheduled an in-person interview at a private meeting space. Participants chose the location of their interview and had the options of meeting in a private office at the university or at a local agency. Each interview began with a research assistant describing the purpose of the study and completing the informed consent process with the participant. Participants had the option of either self-administering the survey via RedCap or having a research assistant verbally administer the survey. All the study participants opted to self-administer the survey via RedCap using a laptop computer in the presence of a research assistant. Each participant completed nine measures and was then invited to participate in a qualitative interview. All the participants were compensated $50 for their participation in our study. Beginning 17 March 2020, all the interviews were conducted via Zoom (Version 5) in order to adhere to COVID-19 public health guidelines and ensure the safety of participants and the research team. Fourteen participants (10.4%) completed their interviews through Zoom. 

### 2.3. Measures

#### 2.3.1. Microaggressions

Exposure to microaggressions was assessed via the LGBQ Microaggressions on Campus Scale [52]. This scale measures two domains of microaggressions: interpersonal microaggressions and environmental microaggressions. The interpersonal microaggressions subscale is comprised of 15 questions pertaining to respondents’ direct experiences of microaggressions (e.g., “Someone told me they were praying for me because they knew or assumed I am lesbian, gay, bisexual, or queer”). The environmental microaggressions subscale is composed of five items (e.g., “In my school/workplace it was OK to make jokes about LGBQ people”). Participants ranked their experiences on a 5-point Likert scale from 0 (never) to 5 (very frequently). Total scores for interpersonal and environmental microaggressions were computed by averaging the responses to the items on each respective subscale. Internal consistency in our sample was excellent for the interpersonal subscale (α = 0.90) and acceptable for the environmental subscale (α = 0.71). 

#### 2.3.2. Psychological Stress

The Brief Symptoms Inventory (BSI), a 53-item scale developed to measure psychological stress, was used to assess participants’ level of anxiety and depression in the last seven days [53]. The anxiety and depression subscales consist of six items each. Examples of items include “Suddenly scared for no reason” (anxiety) and “Feeling hopelessness about the future” (depression). Participants ranked items on a 5-point Likert scale from 0 (not at all) to 4 (extremely). Subscale scores were computed as an average of endorsed items. Internal consistency in our sample was good for both the anxiety (α = 0.87) and depression (α = 0.86) subscale.

#### 2.3.3. Social Support

Perceived level of social support was assessed using the Multidimensional Scale of Perceived Social Support (MSPSS) [54]. The MSPSS is a 12-item scale that assesses social support received from friends, family, and significant other(s). Participants indicated their agreement with each statement (e.g., “I can count on my friends when things go wrong”, “I get the emotional help and support I need from my family”, “There is a special person with whom I can share my joys and sorrows”) on a 7-point Likert scale from 1 (very strongly disagree) to 7 (very strongly agree). The total mean score of all 12 items was used in this study; internal consistency was good in our sample (α = 0.86).

#### 2.3.4. Emotional Comfort from Companion Animals

The 11-item Comfort from Companion Animals Scale (CCAS) was used to assess emotional comfort from companion animals, which is a commonly assessed domain of support from human-animal interaction [55]. The participants responded to items (e.g., “Having a pet gives me something to love”) on a 4-point Likert scale from 1 (strongly disagree) to 4 (strongly agree). A total score was computed by summing all 11 items. The internal consistency in this sample was excellent (α = 0.91).

#### 2.3.5. Covariates

Covariates for this study included race/ethnicity, age, gender identity, and whether the participant was a primary caretaker of their pet within the past 12 months. Participants provided this information in the demographic section of the survey. Additionally, because some interviews took place virtually due to the onset of the COVID-19 pandemic, a dichotomous variable was created to indicate whether study participation occurred before or after the university’s closure due to COVID-19 restrictions. 

#### 2.3.6. Analysis Strategy

All the analyses for this study were conducted using IBM SPSS Statistics (Version 26), and moderation analyses were conducted using PROCESS [56]. We conducted eight simple moderation analyses to determine whether the association between each domain of microaggressions (interpersonal and environmental) and each mental health variable (anxiety and depressive symptoms) varied as a function of emotional comfort from companion animals or human social support (see Figure 1a,b). To better assess the degree to which our findings were influenced by the potential for individuals to seek emotional comfort from companion animals and social support from humans as a response to microaggressions, we also examined separate multiple moderation models that included emotional comfort from companion animals and social support from humans as moderators of the relation between microaggressions and anxiety and depressive symptoms (see Figure 1c). Each model included age, race/ethnicity (0 = racial/ethnic minority, 1 = White, non-Latinx), gender identity (0 = endorsed only cisgender identity, 1 = indicated one or more gender minority identities), whether participation occurred before the onset of the COVID-19 pandemic (=0) or after (=1), and whether the participant reported being the primary caretaker of a dog or cat in the past 12 months (0 = no, 1 = yes) as covariates. 

A post hoc power analysis using the G*Power software [57] was conducted to determine if our sample (*n* = 134) was sufficient to detect a small (f^2^ = 0.02), medium (f^2^ = 0.15), or large (f^2^ = 0.35) incremental effect size [58] at an alpha level of 0.05. The statistical power of our study sample was 0.37 for detecting a small effect and greater than 0.99 for medium to large effects. These findings demonstrate that while we have adequate power (i.e., 0.80) to detect a moderate to large effect size, we have less than adequate power to detect a small effect. Assumptions of normality, linearity, singularity, and homoscedasticity were met. The assumption of multicollinearity was met due to the acceptability of Variance Inflation Factor and tolerance [59].

## 3. Results

The intercorrelations, means, and standard deviations of all variables of interest are displayed in Table 2. Interpersonal microaggressions, environmental microaggressions, and comfort from companion animals scores were significantly related; however, the effect was not strong enough to violate the assumption of multicollinearity. Interpersonal and environmental microaggressions were significantly and positively correlated with depressive and anxiety symptoms. Anxiety was positively correlated with depressive symptoms and emotional comfort from companion animals. All the other correlations were statistically non-significant. Age, race/ethnicity (White vs. racial/ethnic minority), gender minority status (cisgender vs. gender minority), participation after COVID-19 restrictions, and primary caretaker of companion animal were included as covariates in subsequent analyses.

### 3.1. Simple Moderation Analyses

#### 3.1.1. Depressive Symptoms

**Figure 2:**
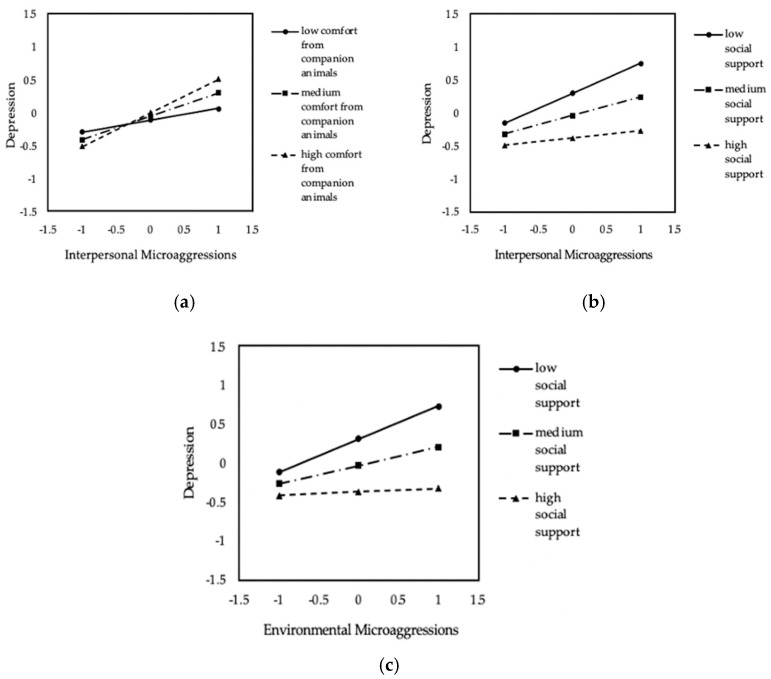
Significant conditional effects of simple moderation (*n* = 134). (**a**) Conditional effect of interpersonal microaggressions on depressive symptoms as a function of comfort from companion animals; (**b**) conditional effect of interpersonal microaggressions on depressive symptoms as a function of social support; (**c**) conditional effect of environmental microaggressions on depressive symptoms as a function of social support. All the variables of interest were standardized.

In our model with depressive symptoms as the dependent variable, we found evidence of a moderation effect of interpersonal microaggressions by emotional comfort from companion animals on depressive symptoms (ΔR^2^ = 0.03, F(1, 125) = 4.78, β = 0.18, t(125) = 2.19, *p* = 0.031). The overall model explains a significant proportion of the variance, R^2^ = 0.16, F(8, 125) = 3.08, *p* = 0.003. As displayed in Figure 2a, the relation between interpersonal microaggressions and depressive symptoms is significant at medium (β = 0.36, t(125) = 4.07, *p* < 0.001) and high levels of comfort from companion animals (β = 0.51, t(125) = 4.50, *p* < 0.001), but not at low levels (β = 0.18, t(125) = 1.52, *p* = 0.131). We did not find evidence of a moderation effect of environmental microaggressions by comfort from companion animals on depressive symptoms (ΔR^2^ = 0.01, F(1, 124) = 1.46, β = 0.10, t(124) = 1.21, *p* = 0.229), and the overall model does not contribute significantly to the variance in depressive symptoms (R^2^ = 0.09, F(8, 124) = 1.54, *p* = 0.150). 

When investigating social support as a moderating variable, social support was a significant moderator of the relation between interpersonal microaggressions and depressive symptoms (ΔR^2^ = 0.03, F(1, 125) = 4.74, β = −0.17, t(125) = −2.18, *p* = 0.031), and the overall model accounts for 26% of the variance in depressive symptoms (F(8, 125) = 5.36, *p* < 0.001). Similarly, the moderation effect of environmental microaggressions by social support on depressive symptoms approached significance, ΔR^2^ = 0.02, F(1, 124) = 3.93, β = −0.19, t(124) = −1.98, *p* = 0.050. The overall model explains 22% of the variance in depressive symptoms, F(8, 124) = 4.32, *p* < 0.001. As shown in Figure 2b,c, the relations between interpersonal and environmental microaggressions and depressive symptoms are significant at low levels of social support (env: β = 0.43, t(124) = 2.94, *p* = 0.004; int: β = 0.46, t(125) = 3.92, *p* < 0.001) and medium levels of social support (env: β = 0.24, t(124) = 2.71, *p* = 0.008; int: β = 0.28, t(125) = 3.39, *p* = 0.001), but not at high levels of social support (env: β = 0.05, t(124) = 0.41, *p* = 0.679; int: β = 0.11, t(125) = 0.94, *p* = 0.350).

#### 3.1.2. Anxiety Symptoms

Social support was not a significant moderator of the relation between interpersonal microaggressions (ΔR^2^ = 0.002, F(1, 125) = 0.31, β = −0.05, t(125) = −0.55, *p* = 0.581) or environmental microaggressions (ΔR^2^ = 0.001, F(1, 124) = 0.09, β = −0.03, t(124) = −0.30, *p* = 0.769) and anxiety symptoms. Although the overall model of interpersonal microaggressions by social support does not explain a significant proportion of the variance in anxiety, R^2^ = 0.09, F(8, 124) = 1.59, *p* = 0.133, the model including environmental microaggressions by social support explains 14% of the variance (F(8, 125) = 2.53, *p* = 0.014). Similarly, we did not find evidence of a moderation effect of interpersonal microaggressions by emotional comfort from companion animals on anxiety (ΔR^2^ = 0.01, F(1, 125) = 0.88, β = 0.08, t(125) = 0.94, *p* = 0.350). However, the overall model explains a significant amount of the variance in anxiety (R^2^ = 0.15, F(8, 125) = 2.84, *p* = 0.006). Comfort from companion animals did not significantly moderate the relation between environmental microaggressions and anxiety (ΔR^2^ = 0.001, F(1, 124) = 0.07, β = 0.02, t(124) = 0.26, *p* = 0.799), nor does it contribute significantly to the variance (R^2^ = 0.10, F(8, 124) = 1.74, *p* = 0.096). 

### 3.2. Multiple Moderation Analyses

#### 3.2.1. Depressive Symptoms

**Figure 3:**
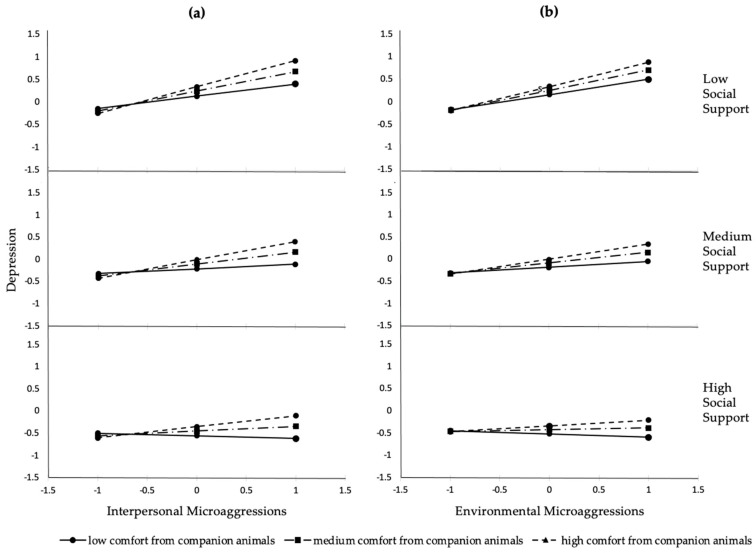
Conditional effects of multiple moderation analyses (*n* = 134). (**a**) Conditional effect of interpersonal microaggressions on depressive symptoms as a function of social support and comfort from companion animals; (**b**) conditional effect of environmental microaggressions on depressive symptoms as a function of social support and comfort from companion animals. All the variables of interest were standardized.

Similar to the results of the simple moderation analyses, multiple moderation analyses indicated that social support was a significant moderator of the relation between interpersonal microaggressions and depressive symptoms when holding comfort from companion animals constant (ΔR^2^ = 0.03, F(1, 123) = 4.40, β = −0.17, t(123) = −2.10, *p* = 0.038). Additionally, comfort from companion animals moderated the relationship between interpersonal microaggressions and depressive symptoms when holding social support constant (ΔR^2^ = 0.03, F(1, 123) = 4.83, β = 0.17, t(123) = 2.20, *p* = 0.030). As shown in Table 3 and Figure 3a, the effect of interpersonal microaggressions on depressive symptoms is significant for those who reported low levels of social support at medium (β = 0.44, t = 3.73, *p* < 0.001) and high (β = 0.58, t = 4.34, *p* < 0.001) levels of comfort from companion animals. The conditional effects also suggest that the relation between interpersonal microaggressions and depressive symptoms is significant for those who reported medium levels of social support at medium (β = 0.27, t = 3.21, *p* = 0.002) and high (β = 0.42, t = 3.85, *p* < 0.001) levels of comfort from companion animals. The effect of interpersonal microaggressions on depressive symptoms is not significantly different from zero among those who reported low comfort from companion animals, regardless of whether one perceived low (β = 0.27, t = 1.94, *p* = 0.055), medium (β = 0.11, t = 0.95, *p* = 0.346), or high (β = −0.06, t = −0.44, *p* = 0.657) levels of social support. Additionally, the effect of interpersonal microaggressions on depressive symptoms is not significantly different from zero among those with medium (β = 0.11, t = 0.93, *p* = 0.355) and high (β = 0.25, t = 1.86, *p* = 0.065) levels of emotional comfort from companion animals and high levels of social support. 

We found that the effect of environmental microaggressions on depressive symptoms was moderated by social support (ΔR^2^ = 0.03, F(1, 122) = 4.27, β = −0.20, t(123) = −2.07, *p* = 0.041), but not by emotional comfort from companion animals (ΔR^2^ = 0.01, F(1, 122) = 1.97, β = 0.11, t(122) = 1.40, *p* = 0.163). The conditional effects (see Figure 3b) suggest that the relation between environmental microaggressions and depressive symptoms is significant for those with low levels of social support across all levels of comfort from companion animals (low: β = 0.33, t = 2.10, *p* = 0.038; medium: β = 0.44, t = 2.86, *p* = 0.005; high: β = 0.54, t = 2.97, *p* = 0.004). Additionally, the relation between environmental microaggressions and depressive symptoms is significant for those who reported medium levels of social support at medium (β = 0.24, t = 2.58, *p* = 0.011) and high (β = 0.33, t = 2.71, *p* = 0.008) levels of comfort from companion animals. There are no significant differences in the effect of environmental microaggressions on depressive symptoms for those who reported medium levels of social support and low levels of comfort from pets (β = 0.13, t = 1.16, *p* = 0.247), or for those with high levels of social support at any level of comfort from companion animals (low: β = −0.07, t = −0.53, *p* = 0.598; medium: β = 0.04, t = 0.33, *p* = 0.744; high: β = 0.13, t =1.00, *p* = 0.317).

#### 3.2.2. Anxiety Symptoms

Although the overall model explains 16% of the variance in anxiety, F(10, 123) = 2.39, *p* = 0.013, we did not find evidence of a moderation effect of comfort from companion animals (ΔR^2^ = 0.01, F(1, 125) = 0.88, β = 0.08, t(125) = 0.94, *p* = 0.350) or social support (ΔR^2^ = 0.002, F(1, 125) = 0.31, β = −0.05, t(125) = −0.55, *p* = 0.581) on the relation between interpersonal microaggressions and anxiety. Similarly, comfort from companion animals (ΔR^2^ = 0.001, F(1, 124) = 0.07, β = 0.02, t(124) = 0.26, *p* = 0.799) and social support (ΔR^2^ = 0.001, F(1, 124) = 0.09, β = −0.03, t(124) = −0.30, *p* = 0.769) were not significant moderators of the relation between environmental microaggressions and anxiety. Moreover, the overall model does not explain a significant proportion of the variance in anxiety (R^2^ = 0.12, F(10, 122) = 1.60, *p* = 0.114).

## 4. Discussion

The purpose of this study was to investigate the potential moderating effects of comfort derived from companion animals and human social support on the relationship between LGBTQ-related microaggressions and mental health outcomes in a sample of LGBTQ+ emerging adults. We expected that experiences of interpersonal and environmental microaggressions would be associated with greater depressive and anxiety symptoms, and that both human social support and comfort derived from companion animals would buffer the impact of these domains of microaggressions on depressive and anxiety symptoms. The results of the simple moderation analyses indicate that human social support is an important factor that mitigates the negative impact of interpersonal and environmental microaggressions on depressive symptoms in LGBTQ+ emerging adults. This confirms our hypothesis and coincides with mounting evidence of the protective impact of human social support on the association between LGBTQ+ minority stressors and depressive symptoms [31,32,33]. The results of the multiple moderation analyses indicate that human social support is a key protective factor that buffers the relationship between microaggressions and depressive symptoms when the levels of emotional comfort from companion animals are held constant. The association between microaggressions and depressive symptoms was not significant when the participants reported high levels of social support, regardless of the level of emotional comfort derived from companion animals. Although we cannot determine the direction of effects from our data, these findings suggest that human social support may serve as a primary protective factor that can disrupt the harmful effect of microaggressions or, alternatively, that people who are less affected by microaggressions are more likely to engage in interpersonal interaction.

Although emotional comfort from companion animals moderated the relationship between interpersonal microaggressions and depressive symptoms, it did not do so in the way that we predicted. The association between interpersonal microaggressions and depressive symptoms in the simple moderation analysis was significantly and positively related at high and medium levels of comfort from companion animals. The results of the multiple moderation analyses further highlight this unexpected relationship, as the association between interpersonal microaggressions and depressive symptoms was significant for those who lacked human social support and concurrently reported high or medium levels of emotional comfort from companion animals. Additionally, the relationship between interpersonal microaggressions and depressive symptoms was approaching significance for participants with low human social support and low comfort from companion animals. These unexpected findings are similar to the results of a study conducted by Antonacopoulos and Pychyl [60], in which dog and cat owners living alone who reported low levels of human social support and high levels of attachment to their companion animals experienced greater depressive symptoms. Antonacopoulos and Pychyl [60] hypothesized that highly attached pet owners may spend more time with their companion animals and, thus, engage in less social interaction with humans, leading them to feel more socially isolated. This is supported by a study conducted by Hartwig and Signal [61], which found that youth who were primary caretakers of their pet reported lower social support scores. Hartwig and Signal [61] also found that attachment to pets was positively associated with loneliness for youth who were primary caretakers. Additionally, evidence from a longitudinal study that found that, after obtaining a pet dog, youths initially received more visits from friends; however, 12 months after getting the dog, youths who were more highly attached to their dog reported spending more time alone and less time with friends and family as compared to youths that were not highly attached to their dog [62]. Our findings further support Antonacopoulos and Pychyl’s [60] hypothesis, in that the association between interpersonal microaggressions and depressive symptoms is significant and positive at high levels of comfort from pets and low and medium levels of social support. Additionally, a study conducted by Barker et al. [63] found that living with a companion animal predicted greater internalizing symptoms among fourth-year undergraduate college students, although the cross-sectional design of this study makes it impossible to make causal inferences. Barker et al. [63] note that it is possible that students experiencing internalizing symptoms are more likely to choose to live with companion animals or, alternatively, the stress of owning a companion animal may contribute to internalizing symptoms. 

Similar to Barker et al.’s [63] study, we are unable to make causal inferences based on the results of our study. It is possible that companion animals may add stress that exacerbates the negative impact of microaggressions on depressive symptoms; however, it is also possible that LGBTQ+ emerging adults who have experienced microaggressions and depressive symptoms seek out more interactions with companion animals that provide them with emotional comfort. Thus, our results add to the existing literature highlighting the complex relationship between human–animal interactions and mental health outcomes and highlight a need for longitudinal studies to clarify the causal nature of these relationships and potentially reciprocal interactions between human–animal interaction, social support, and mental health. 

Our results suggest that both human social support and emotional comfort derived from companion animals play roles in moderating the relationship between microaggressions and depressive symptoms. However, neither social support nor comfort from companion animals moderated the relationship between either domain of microaggressions and anxiety in any of the simple or multiple moderation analyses. These findings suggest that the moderating effect of support from humans and companion animals may not extend to other mental health outcomes, such as anxiety. Further studies are needed to investigate other potential protective factors that may attenuate the impact of microaggressions on symptoms of anxiety in LGBTQ+ emerging adult populations. This may include investigating specific sources of social support (e.g., social support from peers, family, companion animals) or other aspects of human–animal interactions (e.g., stress associated with pet ownership, amount of physical contact or strength of attachment bond) that could be important for reducing anxiety.

### 4.1. Limitations

There are a small number of notable limitations associated with this study. Due to our sample size, we did not have sufficient power to detect small effect sizes. We also did not have a large enough sample to examine differences in relations between microaggressions, depression, and the moderating role of support from humans and pets among individuals with diverse identities (e.g., transgender vs. non-binary, bisexual vs. pansexual, Black vs. Latinx), and instead needed to rely on dichotomous variables for gender and racial majority and minority identities. Because the majority of our sample identified as White, we were additionally unable to investigate the relationship between microaggressions and mental health outcomes amongst intersecting minority identities and did not ask questions specific to other types of microaggressions, such as those due to race/ethnicity, religion, or disability. Additionally, this study did not explore more severe types of victimization, such as exposure to violence, stress associated with living with pets, or other forms of social support (e.g., community-based support or access to identity-affirming social support). Finally, a significant limitation is the cross-sectional design, which does not allow for any causal inferences to be made based on our results.

### 4.2. Future Directions for Research

Microaggressions are only one type of distal stressor experienced by LGBTQ+ individuals. It is important for future studies to distinguish between different forms of discrimination and victimization, as there may be distinct protective factors relevant to specific types of minority stressors experienced. Further, LGBTQ+ emerging adults are a diverse group of people who may identify with multiple marginalized identities. Evidence suggests that individuals who hold multiple marginalized identities experience cooccurring forms of microaggressions based on these intersecting identities, thus putting them at increased risk of poor mental health outcomes [17,64,65]. It is vital that future studies investigate the role that social support may play in influencing the relationship between concurrent forms of distal minority stressors and mental health outcomes in LGBTQ+ individuals who belong to other marginalized groups. Additionally, it may be important to investigate how other domains of human-animal interactions may protect against the impact of polyvictimization. For example, Hawkins et al. [66] found that positive engagement with companion animals buffered the relationship between concurrent exposure to family violence and animal cruelty and internalizing and post-traumatic stress symptoms among pet-owning youth. Future studies should longitudinally examine how social support and relationships with companion animals impact the relationship between various types of stressors on mental health outcomes. In addition, this study focused on examining the relationship between microaggressions and mental health during emerging adulthood and, thus, we are unable to generalize these findings to other periods of development. It is important for future studies to investigate the moderating role of social support and comfort from companion animals on the relationship between microaggressions and mental health for LGBTQ+ individuals in other developmental periods.

## 5. Conclusions

To our knowledge, this is the first study to investigate the potential protective influence of social support from humans and emotional comfort from companion animals on the relationship between multiple forms of microaggressions and mental health symptoms. The results of this study have important implications for future human–animal interaction research and practice with LGBTQ+ populations. Most notably, our findings highlight the important protective role of human social support in mitigating the negative impact of a common form of discrimination, microaggressions, on depressive symptoms. Additionally, emotional comfort derived from companion animals on its own is not sufficient to mitigate, and could potentially exacerbate, the harmful impact of microaggressions on mental health symptoms in LGBTQ+ emerging adult populations. Our findings underscore the critical need for future researchers to use longitudinal methods to clarify the causal relationship between emotional comfort from companion animals and mental health outcomes. Our study highlights the importance of inquiring about social support and relationships with pets when working with LGBTQ+ emerging adult populations.

## Figures and Tables

**Table 1:** Demographic information (*n* = 134).

Variable Name	Variable Categories	Frequency (%)
Racial/Ethnic Identity	Arab/Arab American	1 (0.7%)
	Asian/Asian American	2 (1.5%)
	Black/African American	20 (14.9%)
	Latina/Latino/Latinx	8 (6.0%)
	Multiracial/Mixed Race	17 (12.7%)
	South Asian/Pacific Islander	1 (0.7%)
	White	84 (62.7%)
	Prefer to self-describe	1 (0.7%)
Gender Identity	Agender	4 (3.0%)
	Cisgender man	10 (7.5%)
	Cisgender woman	58 (43.3%)
	Genderfluid	2 (1.5%)
	Genderqueer	5 (3.7%)
	Nonbinary	11 (8.2%)
	Transgender man	16 (11.9%)
	Transgender woman	2 (1.5%)
	Multiple Identifications	20 (14.9%)
	Not sure/questioning/prefer to self-describe	6 (4.5%)
Sexual Orientation	Asexual	2 (2.5%)
	Bisexual	30 (22.4%)
	Demisexual	2 (0.7%)
	Gay	11 (8.2%)
	Lesbian	16 (11.9%)
	Pansexual	13 (9.7%)
	Queer	19 (14.2%)
	Straight/heterosexual	2 (1.5%)
	Multiple Identifications	40 (29.9%)
Pet Type—Lived with ^1^	Bird	2 (1.5%)
	Cat	80 (59.7%)
	Dog	90 (67.2%)
	Lagomorph	9 (6.7%)
	Rodent	7 (5.2%)
	Other (e.g., fish, tarantula, reptile)	16 (11.9%)
Pet Type—Primary Caretaker ^2^	Bird	2 (1.5%)
	Cat	40 (29.9%)
	Dog	27 (20.1%)
	Lagomorph	4 (3.0%)
	Rodent	6 (4.5%)
	Other (e.g., fish, reptile)	13 (9.7%)

^1^ Participants were able to report information on a maximum of three pets they have lived with in the past year. These categories are not mutually exclusive. ^2^ Participants reported whether or not they identified themselves as the primary caretaker of each specified pet. These categories are also not mutually exclusive.

**Table 2:** Intercorrelations, unstandardized means, and standard deviations (*SD*) for constructs of interest (*n* = 134).

Variable	*M*	*SD*	1	2	3	4	5	6	7	8	9	10	11
1. Age	19.31	1.12	-										
2. White ^1^			−0.01	-									
3. Gender Minority Status ^2^			0.13	0.05	-								
4. COVID ^3^			0.10	−0.04	−0.14	-							
5. Primary Caretaker ^4^			0.02	0.11	0.13	−0.07	-						
6. Interpersonal Microaggressions	2.54	1.02	−0.03	0.06	0.01	−0.03	0.30 ***	-					
7. Environmental Microaggressions	3.82	0.89	−0.16	0.04	−0.13	0.02	0.18 *	0.56 ***	-				
8. Depressive Symptoms	1.88	0.93	0.03	−0.01	0.12	0.02	0.07	0.34 ***	0.23 **	-			
9. Anxiety	1.90	0.85	0.10	0.07	0.14	−0.02	0.16	0.32 ***	0.19 ***	0.60 ***	-		
10. Social Support	5.28	0.99	−0.04	0.03	−0.08	−0.09	−0.20 *	−0.26 **	−0.19 *	−0.39 ***	0.17	-	
11. Comfort from Companion Animals	40.34	4.25	0.07	0.05	−0.15	−0.11	0.35 ***	0.29 **	0.30 ***	0.06	0.20 *	0.06	-

* *p* < 0.05. ** *p* < 0.01. *** *p* < 0.001. ^1^ 0 = other and 1 = White. ^2^ 0 = cisgender and 1 = gender minority. ^3^ 0 = participated before and 1 = participated after COVID-19 restrictions were established. ^4^ 0 = not a primary caretaker and 1 = primary caretaker of dog and/or cat in the past 12 months.

**Table 3:** Conditional effects of interpersonal and environmental microaggressions on depressive symptoms at different levels of social support and comfort from companion animals (*n* = 134).

Variable	Social Support	Comfort from Companion Animals	β	SE	t	*p*	95% CI	
							LL	UL
Interpersonal Microaggressions x Depressive Symptoms		Low	0.27	0.14	1.94	0.05	−0.01	0.55
	Low	Medium	0.44	0.12	3.73	<0.001	0.21	0.67
		High	0.58	0.13	4.34	<0.001	0.32	0.85
	Medium	Low	0.11	0.11	0.95	0.35	−0.12	0.33
		Medium	0.27	0.08	3.21	0.002	0.10	0.44
		High	0.42	0.11	3.85	<0.001	0.20	0.63
	High	Low	−0.06	0.13	−0.95	0.66	−0.33	0.21
		Medium	0.10	0.11	0.93	0.35	−0.12	0.33
		High	0.25	0.13	1.86	0.06	−0.02	0.51
Environmental Microaggressions x Depressive Symptoms	Low	Low	0.33	0.16	2.10	0.04	0.02	0.65
		Medium	0.44	0.15	2.86	0.01	0.14	0.75
		High	0.54	0.18	2.97	0.004	0.18	0.89
	Medium	Low	0.13	0.11	1.16	0.25	−0.09	0.35
		Medium	0.24	0.09	2.58	0.01	0.06	0.42
		High	0.33	0.12	2.71	0.01	0.09	0.58
	High	Low	−0.07	0.14	−0.53	0.60	−0.35	0.20
		Medium	0.04	0.11	0.33	0.74	−0.19	0.26
		High	0.13	0.13	1.00	0.32	−0.13	0.39

Note. CI = confidence interval. LL = lower level. UL = upper level.

## Data Availability

The data presented in this study are available on request from the second author. The data are not publicly available due to potentially identifiable information in the dataset.
